# Redundancy in Innate Immune Pathways That Promote CD8^+^ T-Cell Responses in AAV1 Muscle Gene Transfer

**DOI:** 10.3390/v16101507

**Published:** 2024-09-24

**Authors:** Ning Li, Sandeep R. P. Kumar, Di Cao, Maite Munoz-Melero, Sreevani Arisa, Bridget A. Brian, Calista M. Greenwood, Kentaro Yamada, Dongsheng Duan, Roland W. Herzog

**Affiliations:** 1 Department of Pediatrics, Herman B Wells Center for Pediatric Research, Indiana University, Indianapolis, IN 46202, USA; gordonln@outlook.com (N.L.); caodi@iu.edu (D.C.); mamelero@iu.edu (M.M.-M.); sarisa@iu.edu (S.A.); bribrian@iu.edu (B.A.B.); caligree@iu.edu (C.M.G.); yamadak@iu.edu (K.Y.); 2 Department of Molecular Microbiology and Immunology, School of Medicine, University of Missouri, Columbia, MO 65211, USA; duand@missouri.edu

**Keywords:** adeno-associated virus, CD8 T cell, TLR9, IL-1, muscle

## Abstract

While adeno-associated viral (AAV) vectors are successfully used in a variety of in vivo gene therapy applications, they continue to be hampered by the immune system. Here, we sought to identify innate and cytokine signaling pathways that promote CD8^+^ T-cell responses against the transgene product upon AAV1 vector administration to murine skeletal muscle. Eliminating just one of several pathways (including DNA sensing via TLR9, IL-1 receptor signaling, and possibly endosomal sensing of double-stranded RNA) substantially reduced the CD8^+^ T-cell response at lower vector doses but was surprisingly ineffective at higher doses. Using genetic, antibody-mediated, and vector engineering approaches, we show that blockade of at least two innate pathways is required to achieve an effect at higher vector doses. Concurrent blockade of IL-1R1 > MyD88 and TLR9 > MyD88 > type I IFN > IFNaR pathways was often but not always synergistic and had limited utility in preventing antibody formation against the transgene product. Further, even low-frequency CD8^+^ T-cell responses could eliminate transgene expression, even in MyD88- or IL-1R1-deficient animals that received a low vector dose. However, we provide evidence that CpG depletion of vector genomes and including TLR9 inhibitory sequences can synergize. When this construct was combined with the use of a muscle-specific promoter, transgene expression in muscle was sustained with minimal local or systemic CD8^+^ T-cell response. Thus, innate immune avoidance/blockade strategies by themselves, albeit helpful, may not be sufficient to prevent destructive cellular responses in muscle gene transfer because of the redundancy of immune-activating pathways.

## 1. Introduction

Adeno-associated viral (AAV) vectors are widely used in clinical gene therapy for in vivo transfer of therapeutic genes to various organs and cell types. These vectors contain a DNA genome (single-stranded or self-complementary) that is devoid of viral coding sequences and packaged into a protein capsid. Tissue tropism and efficiency of gene transfer to different cell types is determined by the choice of capsid, route of administration, and vector dose. Transduction of skeletal muscle fibers by intramuscular (IM) administration has undergone clinical trials for treatment of different forms of muscular dystrophy [1,2,3] and for systemic protein delivery (for hemophilia B and α_1_-antitrypsin deficiency, for example) [4,5] and also formed the basis for the first gene therapy product approved in the Western world (Glybera for systemic delivery of lipoprotein lipase to treat lipoprotein lipase deficiency) [6]. This approach has been further clinically tested for passive immunization based on the delivery of antibodies against HIV [7,8,9] and is in development for delivery of Cas9 and gRNA (guide RNAs) for CRISPR-mediated gene editing of dystrophic muscle [10,11]. Vascular delivery for the widespread correction of muscle forms the basis for a recently studied gene therapy for patients with Duchenne Muscular Dystrophy (DMD) [12] and is in advanced clinical testing for a variety of neuromuscular diseases [13]. Muscle disorders that could benefit from direct IM injection include tibial muscular dystrophy, facioscapulohumeral muscular dystrophy, and oculopharyngeal muscular dystrophy.

Although AAV vectors elicit less innate immunity and are less efficient in activating CD8^+^ T-cell responses compared to many other vector systems, immune responses are nonetheless a major hurdle for AAV-based gene therapies, especially when given at high vector doses [14,15,16,17,18]. The IM route may increase immunogenicity (and is thus often used for vaccine delivery), and the viral particles carry pathogen-associated molecular patterns (PAMPs) that can be recognized by innate pattern recognition receptors. This is particularly well documented for sensing the AAV genome by the endosomal DNA receptor TLR9 [19,20]. Initially discovered by Yang and colleagues, plasmacytoid dendritic cells (pDCs) produce type I IFN upon activation of the TLR9-MyD88 signaling pathway [19]. These events may link innate immune sensing of the AAV genome to the induction of an antigen-specific immune response. Priming of CD8^+^ T cells occurs through the cooperation of pDCs and conventional DCs (cDCs), which present capsid or transgene product antigens via MHC-I and activate CD8^+^ T cells upon sensing of type I IFN with their IFNaR receptors [20,21]. Antigen presentation is likely carried out by XCR1^+^ cDCs, which are particularly efficient in cross-presentation via MHC-I and are known to serve as a platform for sequential interactions with CD4^+^ and CD8^+^ T cells [22,23]. CD4^+^ T-helper cells provide co-stimulation that, in addition to cytokine signaling, is critical for the priming of CD8^+^ T cells [21,24,25]. In contrast to mammalian DNA, viral and bacterial genomes lack CpG methylation [26,27]. Such unmethylated CpG motifs are particularly potent stimulators of TLR9 [22,27,28]. Thus, a common strategy to reduce the risk of CD8^+^ T-cell activation in AAV gene transfer is to synthesize gene constructs that lack CpG motifs (“CpG depletion” strategy) [29,30,31,32]. Alternatively, sequences can be included in the AAV vector genome that are inhibitory to TLR9 (TLR9i) [33]. TLR9 signaling also mediates early innate immune responses in the target organ that can occur within hours after vector administration [34]. However, TLR9 is less critical for antibody formation (albeit some TLR9 ligands may enhance antibody formation by increasing recruitment of monocyte-derived dendritic cells (moDCs), as we showed by co-administration of vector and oligodeoxynucleotides to skeletal muscle) [35,36,37].

Our recent study in hepatic gene transfer uncovered a TLR9-independent pathway that promotes CD8^+^ T-cell responses against the transgene product via IL-1R1-MyD88 signaling [25,38]. This mechanism involves infiltration of IL-1α/β-producing pDCs and formation of pDC/XCR1^+^ DC/CD8^+^ T cell co-clusters in the liver. While both IL-1α and IL-1β are contributing, CD8^+^ T-cell activation was found to be independent of inflammasomes. IL-1α serves as an “alarmin” in responses against pathogens, may be cell-bound or extracellular, and does not require proteolytic processing to be active. Recombinant IL-1R antagonist (anakinra) is an approved anti-inflammatory drug for blockade of the IL-1R1 receptor. Whether this pathway is important for muscle gene transfer has been unknown.

CD8^+^ T-cell responses against transgene products have been documented in some DMD and α_1_-antitrypsin-deficient patients who received muscle-directed AAV gene transfer [3,39,40,41,42,43]. More recently, we showed that potent CD8^+^ T-cell responses directed against a Cas9 transgene product eliminated AAV-transduced muscle fibers in canine models [44]. Route of administration, vector design and dose, as well as the underlying mutation and polymorphisms in the affected gene, impact the risk of such responses in gene replacement therapy [13,31,34,45,46]. Inflammation or tissue damage may be sources of Damage Associated Molecular Patterns (DAMPs) that contribute to T-cell activation. In this study, we were surprised to find induction of transgene product-specific CD8^+^ T cells upon IM administration of AAV vector in TLR9 and IFNaR-deficient mice at frequencies similar to those in wild-type (WT) mice when higher vector doses were used. Further investigations showed that multiple pathways, including IL-1 signaling, have redundant roles in promoting CD8^+^ T-cell responses. Eliminating a single pathway was sufficient to substantially reduce the response at lower but not higher vector doses. We document the utility and limitations of blocking innate immune pathways to prevent CD8^+^ T-cell responses in muscle-directed gene transfer.

Our experiments utilized AAV serotype 1, which was the first capsid shown to direct superior transduction of skeletal muscle and was also the capsid of the first gene therapy vector to achieve regulatory approval in the Western world (Glybera, which was administered by IM injection to patents with familial lipoprotein lipase deficiency) [6,47,48]. AAV1 has since been evaluated in multiple clinical trials in skeletal muscle, cardiac, and diaphragm gene transfer for the treatment of a variety of inherited and acquired diseases [1,2,49,50,51,52,53,54].

## 2. Materials and Methods

### 2.1. AAV Vector Production

All AAV vectors were of serotype 1. AAV expression cassette for chicken ovalbumin (OVA) was under the control of either cytomegalovirus (CMV) promoter, or a combination of CMV immediate early gene (IE) 1 enhancer and human elongation factor-1α (EF1α) promoter, or the muscle-specific creatine kinase 8 (ck8) promoter (which was synthesized according to information in US patent 10479821B2). The CpG-free OVA expression cassette (with CMV enhancer/EF1α promoter) was purchased from InvivoGen (San Diego, CA, USA). All AAV vectors were packaged using triple transfection of HEK293 T cells, purified using iodixanol gradient centrifugation and titrated by quantitative polymerase chain reaction (PCR) as previously reported [55].

### 2.2. Mouse Strains and Experiments

C57BL/6J WT, C57BL/6NJ WT, B6N.129S1-Tlr3*^tm1Flv^*/J (TLR3^−/−^), B6.129P2(SJL)-Myd88*^tm1.1Defr^*/J (MyD88^−/−^), B6.129S7-IL1r1*^tm1Imx^*/J (IL-1R1^−/−^), C57BL/6J-Tbk1*^em10Lutzy^*/J (TBK1^−/−^), B6.Cg-Ifih1*^tm1.1Cln^*/J (MDA5^−/−^), C57BL/6NJ-Rigi*^em1(IMPC)J^*/Mmjax (RIG-I^−/−^), C57BL/6J-Ticam1*^Lps2^*/J (TRIF^−/−^), C57BL/6J-Sting1*^gt^*/J (STING^−/−^) mice were purchased from Jackson Laboratories (Bar Harbor, ME, USA). TLR9^−/−^ [20], CD11c^cre^ × MyD88^fl/fl^ (CD11c-MyD88^−/−^) [20] and CD11c^cre^ × IFNaR^fl/fl^ (CD11c-IFNaR^−/−^) mice were bred in house (all on C57BL/6J background) [21]. All mice were 6–8 weeks old at the start of the experiment, and each experimental group consisted of five mice. Mice were housed in the laboratory animal resource center facility at Indiana University (IU), Indianapolis. These studies were conducted under protocols 21017 (approved on 15 April 2021) and 23173 (approved on 27 March 2024) by the Institutional Animal Care and Use Committee (IACUC) of Indiana University School of Medicine.

Mice were injected into the quadriceps muscle with either 2 × 10^10^ vg or 2 × 10^11^ vg of AAV vector in total volume of 50 μL. Type I IFN signaling was blocked by the administration of 250 μg of αIFNAR-1 (MAR1-5A3) antibody via intraperitoneal (IP) route [21]. IL-1 signaling was prevented by neutralization of cytokines IL-1α and IL-1β, with co-administration of αIL-1α (clone ALF-161) and αIL-1β (clone B122) antibodies (200 μg each) via IP route [25,56]. Control mice received an equal amount of isotype antibody. All the antibody treatments were initiated one day prior to vector administration and were continued for up to 6 weeks, 2 times/week. All in vivo blocking/neutralization antibodies were from BioXcell (Lebanon, NH, USA).

### 2.3. Flow Cytometry

Peripheral blood mononuclear cells (PBMCs) were pretreated with TruStain FcX anti-mouse CD16/CD32 (Fc receptor block; BioLegend, San Diego, CA, USA). CD8^+^ T cells were distinguished from CD4^+^ T cells via a combination of CD3 and CD8 antibodies. OVA-specific CD8^+^ T cells were assessed using MHC class I tetramer iTAg (H2-Kb-SIINFEKL; MBL International, Woburn, MA, USA) as per the manufacturer’s instructions. OVA-specific CD8^+^ T cells were further analyzed for their activation status using antibodies against CD44, CD62L and CD107 surface markers. PBMCs were surface stained at room temperature for 20 min. PBMCs were washed, and red blood cells were lysed with VersaLyse (Beckman Coulter, Brea, CA, USA). PBMCs were acquired on Attune (Invitrogen, Waltham, MA, USA), and analysis was performed with FCS Express 7 (De Novo Software, Pasadena, CA, USA). All the antibodies were purchased from Biolegend (San Diego, CA, USA) unless stated otherwise.

### 2.4. Immunohistochemistry

AAV-injected muscles were frozen, as previously reported [57]. Briefly, excised muscles were mounted on a 3-inch dowel using 10% (*w*/*v*) gum tragacanth (Sigma, St. Louis, MO) in PBS. The dowel was then gently inverted and submerged into a 15.0 mL conical tube that contained 5.0 mL of pre-chilled 2-methylbutane (isopentane; GFS chemicals, OH). The 15.0 mL tubes were then stored at −20 °C until used. Frozen muscles were sectioned (∼10 μm) using a cryostat (Leica Biosystems, Deer Park, IL, USA). Muscle sections were mounted on poly-lysine-coated slides, and immunohistochemistry was performed as described previously [46]. Sections were stained for OVA expression using rabbit α-OVA (Abcam, Cambridge, UK) and CD8^+^ T (clone 53-6.7; Biolegend, San Diego, CA, USA) cell infiltration. Fluorophore conjugated α-rabbit and α-rat secondary antibodies were from Abcam. Sections were mounted with ProLong Diamond Antifade (Life Technologies, Carlsbad, CA, USA) mounting medium containing DAPI (2-(4-amidinophenyl)-1H-indole-6-carboxamidine). Fluorescence images were captured with a Zeiss Axio Observer 7 microscope (Zeiss, Jena, Germany) and analyzed with ZEN Blue edition 3.5 software.

### 2.5. Antibody Assays

Mouse plasma was analyzed for OVA-specific IgG2c antibodies as previously published [34,35]. An amount of 10 μg/mL of OVA was used for coating ELISA wells. Plasma was used at 1:40 dilution in dilution buffer (5% bovine serum albumin and 0.05% Tween 20 in 1X phosphate buffered saline). HRP-conjugated goat anti-mouse IgG2c (SouthernBiotech, Birmingham, AL, USA) was used as the detection antibody. Data were acquired using an Epoch 2 microplate spectrophotometer (BioTek, Winooski, VT, USA) with GEN 5-3.11 software.

### 2.6. Statistical Analysis

All data are presented as means ± standard error of the mean (SEM). Data were analyzed using GraphPad Prism 10.3.1 software (San Diego, CA, USA). Multiple unpaired *t*-tests with Holm–Šídák post hoc correction were used, and *p*-values of ≤0.05 were considered significant and are indicated as (* *p* ≤ 0.05, ** *p* < 0.01, *** *p* < 0.001, and **** *p* < 0.0001).

## 3. Results

### 3.1. Single-Stranded AAV Vector Induces TLR9-Independent CD8^+^ T-Cell Response Following High-Dose Intramuscular Injection

**Figure 1:**
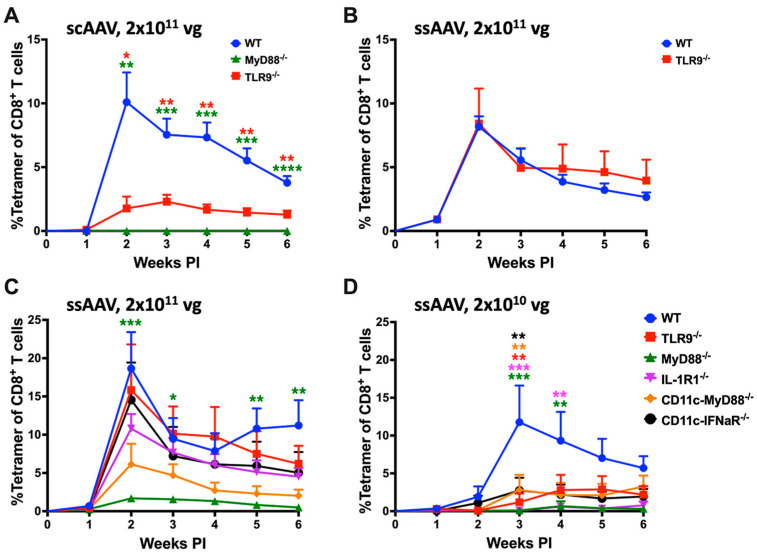
AAV genome conformation and vector dose dictate innate sensing and cellular response to AAV-encoded transgene product in muscle. (**A**) Kinetics of OVA-specific CD8^+^ T-cell response in C57BL/6 − WT, TLR9^−/−^ and MyD88^−/−^ mice following intramuscular gene transfer with a high dose (2 × 10^11^ vg) of scAAV1 vector. (**B**) Kinetics of OVA-specific CD8^+^ T-cell response in C57BL/6J − WT and TLR9^−/−^ mice following intramuscular gene transfer with a high dose (2 × 10^11^ vg) of the ssAAV vector. (**C**,**D**) Kinetics of OVA-specific CD8^+^ T-cell response in C57BL/6J − WT and knockout mice lacking specific innate sensor (either universally or in a particular cell type), following intramuscular gene transfer with high (2 × 10^11^ vg; (**C**)) and low (2 × 10^10^ vg; (**D**)) dose of the ssAAV1 vector. Each experimental group consisted of five mice. Data are presented as mean ± SEM. The level of statistical significance between WT and knockout mice is indicated as * *p* ≤ 0.05, ** *p* < 0.01, *** *p* < 0.001, and **** *p* < 0.0001. Non-significant differences are not indicated.

The objective of this study was to identify innate immune pathways that drive the activation of transgene product-specific CD8^+^ T cells in AAV gene transfer to skeletal muscle. We utilized the AAV1 serotype, which efficiently transduces muscle and has been extensively tested in clinical settings. The choice of OVA as transgene allowed us to track OVA-specific CD8^+^ T cells over time in the periphery with MHC class I tetramer. Sensing of the AAV genome (in particular unmethylated CpG motifs, which are typical for viral DNA) by TLR9 is widely viewed as the critical innate signal for priming of CD8^+^ T cells in the context of AAV gene transfer. In earlier studies, we found that vectors with self-complementary genomes (scAAV) more strongly activate TLR9-dependent immune responses than traditional single-stranded (ssAAV) vectors [34]. Consistent with prior literature, IM administration of 2 × 10^11^ vector genomes (vg) of scAAV1-CMV-OVA induced a strong CD8^+^ T-cell response against OVA in WT mice that peaked (~10% of total CD8^+^ T cells) at 2 weeks following vector administration while TLR9^−/−^ mice had a substantially lower (~2% of total CD8^+^ T cells) response and MyD88^−/−^ mice failed to mount a CD8^+^ T-cell response against the transgene product (Figure 1A) [19,22,29,35,58]. Using a ssAAV1-CMV-OVA vector resulted in an equally high CD8^+^ T-cell response in WT mice (Figure 1B). To our surprise, TLR9^−/−^ mice showed the same level of CD8^+^ T-cell induction as WT mice when the ssAAV1-CMV-OVA vector was administered (Figure 1B).

### 3.2. TLR9, Type I IFN, and IL-1R1 Are Crucial for Maximum CD8^+^ T Cell Response to the Transgene Product at Lower but Not at Higher Vector Doses

Given this unexpected result, we decided to investigate the innate sensing requirements of the ssAAV1-CMV-OVA vector in influencing the transgene product-specific cellular responses. We injected either 2 × 10^10^ vg (low) or 2 × 10^11^ vg (high) of the ssAAV1-CMV-OVA vector into the quadriceps muscles of C57BL/6J − WT, TLR9^−/−^ and MyD88^−/−^ mice (*n* = 5) and evaluated the frequencies of circulating OVA-specific CD8^+^ T cells in PBMCs as a function of time (Figure 1C,D and Supplementary Figure S1A,B). OVA-specific CD8^+^ T-cell responses were again similar in TLR9^−/−^ and WT mice at the high dose, while TLR9^−/−^ mice showed a substantial reduction in the response at the low-vector dose (Figure 1C,D). Mice deficient in MyD88 had minimal responses at both vector doses (~2% at the high dose and delayed response of <1% at the low dose; Figure 1C,D). In WT mice, the peak of the response (average of ~12% of total CD8^+^ T cells) at the low-vector dose is observed at 3 weeks, one week later compared to the high-vector dose (average of ~19% at week 2; Figure 1B–D). Given our recent discovery that mice lacking IL-1R1 fail to develop CD8^+^ T-cell responses against OVA in hepatic AAV gene transfer [25], we investigated the role of IL-1 signaling in transgene product-specific cellular response in muscle gene transfer. We evaluated the frequencies of circulating OVA-specific CD8^+^ T cells in IL-1R1^−/−^ mice following intramuscular (IM) delivery of either 2 × 10^10^ vg (low) or 2 × 10^11^ vg (high) of the ssAAV1-CMV-OVA vector. At the high-vector dose, IL-1R1^−/−^ mice had a modest reduction in OVA-specific CD8^+^ T cells (no statistical difference) compared to WT and TLR9^−/−^ mice (Figure 1C). However, at the low dose, a significant reduction in OVA-specific CD8^+^ T cells was observed in comparison to WT mice, which was also lower than in TLR9^−/−^ mice (Figure 1D). Combined, these results suggest a redundant role of TLR9 and IL-1R1 pathways in activating CD8^+^ T cells against the transgene product. Both pathways are required for a maximal response at low-vector doses, while at high doses, either pathway may be sufficiently active to drive the response (or, alternatively, replaced by a third pathway). Interestingly, at the 2 × 10^11^ vg dose, IL-1R1^−/−^ had fewer OVA-specific CD8^+^ effector T (Teff) cells and a higher portion of T effector memory cells (TEM) at 2 weeks when compared to WT and TLR9^−/−^ mice, which was reversed at subsequent time points (Supplementary Figure S1C). While the magnitude of the CD8^+^ T-cell response is identical in the absence of the IL-1R, the Teff response is delayed and then prolonged.

Previous studies have shown that TLR9 activation by the AAV genome in pDCs results in type I IFN production, which is sensed by the IFNaR receptor on cDCs [20,21]. Consistent with this pathway and the above-described results in TLR9^−/−^ animals, CD11c-IFNaR^−/−^ (only CD11c^+^ cells lack type I IFN receptor) mice show a significant reduction in the CD8^+^ T-cell response at the low (2 × 10^10^ vg) but not the high (2 × 10^11^ vg) vector dose (Figure 1C,D). We have previously shown that IFNaR but not MyD88 expression is required in cDCs in TLR9-dependent responses to AAV [21]. If IL-1R1 signaling is required in cDCs, then the [MyD88^fl/fl^ × cre-CD11c] mice (which lack MyD88 in CD11c^hi^ cDCs but not in pDCs) should have substantially reduced CD8^+^ T-cell responses to OVA. This is indeed what we observed at the low-vector dose (Figure 1D). Although not reaching statistical significance, the average response was also reduced (by ~3-fold) compared to WT mice at the high-vector dose (Figure 1C).

**Figure 2:**
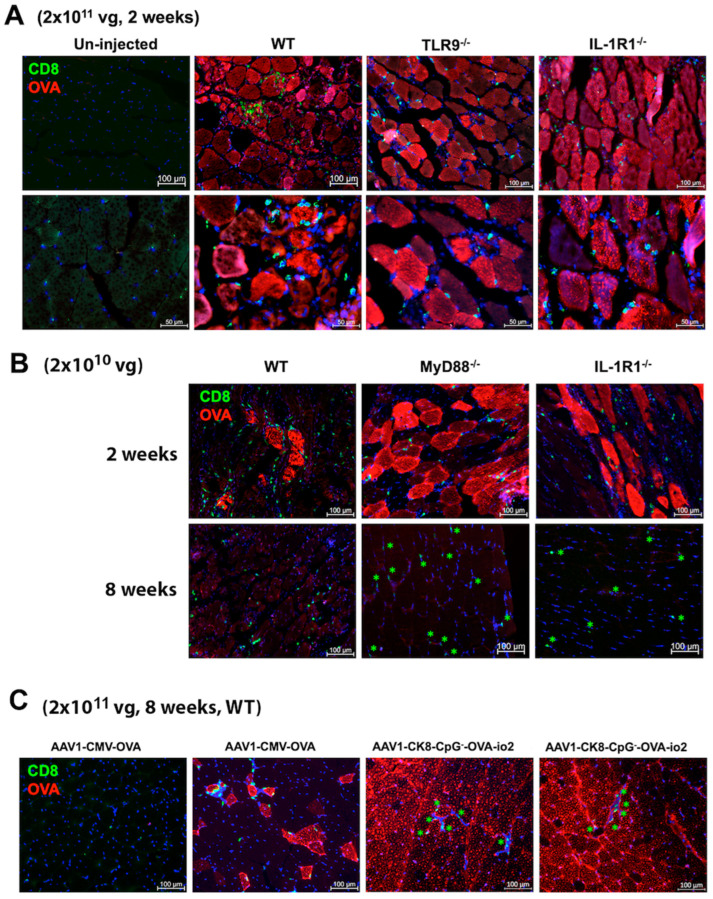
CD8^+^ T cells infiltrate and surround muscle fibers expressing OVA. (**A**) Muscle sections from C75BL/6J − WT, TLR9^−/−^, and IL-1R1^−/−^ mice showing OVA-expressing muscle fibers (red) and CD8^+^ T cells (green) infiltrate 2 weeks after administration of a high dose (2 × 10^11^ vg) of the ssAAV1-CMV-OVA vector. Also shown is the un-injected WT muscle (negative control). Images were generated using a 20× (**top panel**) or 40× (**lower panel**) objective. (**B**) Images (20×) of muscle sections of C57BL/6J − WT, MyD88^−/−^, and IL-1R1^−/−^ mice 2 or 8 weeks after administration of low dose (2 × 10^10^ vg) of the ssAAV1-CMV-OVA. (**C**) Images (20×) of muscle sections of C57BL/6J − WT 8 weeks after administration of a high dose (2 × 10^11^ vg) of the ssAAV1-CMV-OVA (left two images) or ssAAV1-ck8-CpG^−^-OVA-io2 (right two images) vector. In some images, the CD8^+^ T cells are indicated with green asterisks. Images are representative of transduced muscle from *n* = 3 mice/experimental group.

To assess the effect of CD8^+^ T-cell responses on OVA expression in muscle fibers, we performed immunohistochemistry on muscle sections from ssAAV1 injected mice (*n* = 3/experimental group). Transduced muscles from the WT, TLR9^−/−^, and IL-1R1^−/−^ mice all showed both widespread OVA expression targeted by CD8^+^ T-cell infiltration two weeks after high-dose vector administration (Figure 2A). Therefore, the systemic T-cell response correlated with a local immune response in the muscle. When we stained muscles from WT, MyD88^−/−^ or IL-1R1^−/−^ mice after administration of the low-dose vector, we found similar levels of muscle fiber transduction, accompanied by CD8^+^ T-cell infiltration in all three strains at 2 weeks, while some residual infiltrating CD8^+^ T cells but no OVA-expressing fibers were detected at 8 weeks (Figure 2B). Therefore, even low-frequency CD8^+^ T-cell responses were likely sufficient to target and eliminate transgene expression.

### 3.3. Potential Role for dsRNA Sensing

**Figure 3:**
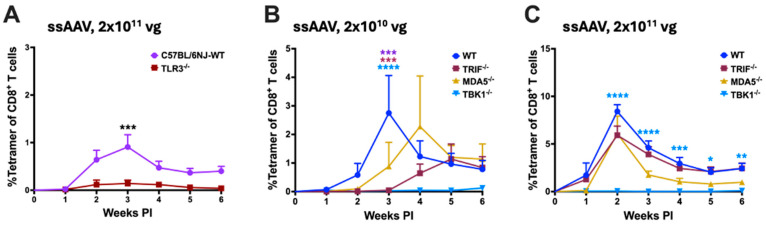
Differential requirements for TLR3 and cytoplasmic nucleic acid sensors at different vector doses. (**A**) Kinetics of OVA-specific CD8^+^ T-cell response in C57BL/6NJ − WT and TLR3^−/−^ following intramuscular gene transfer with a high dose (2 × 10^11^ vg) of the ssAAV1 vector. (**B**,**C**) Kinetics of OVA-specific CD8^+^ T-cell response in C57BL/6J − WT and knockout mice lacking specific cytoplasmic nucleic acid innate sensor, following intramuscular gene transfer with low (2 × 10^10^ vg; (**B**) and a high dose (2 × 10^11^ vg; (**C**)) of the ssAAV1 vector. Each experimental group consisted of five mice. Data are presented as mean ± SEM. Significant differences between WT and knockout mice are denoted as follows: the level of statistical significance is indicated as * *p* ≤ 0.05, ** *p* < 0.01, *** *p* < 0.001, and ****p < 0.0001. Non-significant differences are not indicated.

Since blocking either TLR9 or IL-1R1 signaling did not entirely prevent transgene product-specific cellular response, we probed if alternative innate immune sensors could drive these T-cell responses. First, we assessed the role of the endosomal innate sensor TLR3, a sensor of double-stranded (ds) RNA, on transgene product-specific cellular responses. We compared the OVA-specific CD8^+^ T-cell response in WT- and TLR3-deficient C57BL/6NJ mice following IM administration of AAV1 vector encoding OVA. For unknown reasons, C57BL/6NJ mice have a weaker CD8^+^ T-cell response following AAV gene transfer than C57BL/6J mice and thus require a dose of 2 × 10^11^ vg to yield a measurable response [25]. WT C57BL/6NJ mice showed a modest response after IM administration of 2 × 10^11^ vg, which was nearly absent in TLR3^−/−^ mice (Figure 3A). Unlike most TLRs, which signal through MyD88, TLR3 utilizes TRIF (TIR-domain-containing adaptor protein-inducing interferon-β) as its cytoplasmic adaptor. To further confirm the findings in TLR3^−/−^ mice, we assessed the frequency of OVA-specific CD8^+^ T-cells in TRIF^−/−^ mice following IM administration of the ssAAV1 vector. Compared to WT C57BL/6J mice, TRIF^−/−^ mice had a significantly attenuated CD8^+^ T-cell response against OVA at the low dose (2 × 10^10^ vg), while no difference was seen at the high dose (2 × 10^11^ vg; Figure 3B,C). Thus, TLR3-TRIF signaling may play a role when using limited vectors.

Cytoplasmic RNA sensors, RIG-I and MDA5, have recently been shown to be critical in the innate sensing of AAV [59]. To interrogate a potential role for cytoplasmic sensors of dsRNA, we assessed OVA-specific CD8^+^ T-cell responses in mice deficient in MDA5 or RIG-I pathways (MDA5^−/−^ and DDX58^−/−^, respectively). Lack of MDA5 only slightly delayed the response at a low-vector dose (2 × 10^10^ vg), whereas at a high-vector dose (2 × 10^11^ vg), OVA-specific CD8^+^ T-cell response was comparable to WT mice (Figure 3B,C). Mice that were deficient for RIG-I signaling (DDX58^−/−^ C57BL/6NJ mice) had robust CD8^+^ T-cell responses when compared to WT C57BL/6NJ mice (compare Supplementary Figure S2 and Figure 3A). In summary, we find evidence for a role of TLR3-TRIF signaling but not MDA5 or RIG-I in CD8^+^ T-cell response to OVA in AAV muscle gene transfer.

Finally, we evaluated the role of cytoplasmic dsDNA sensing in the activation of transgene product-specific CD8^+^ T-cell response following AAV gene transfer in muscle. For this, we utilized C57BL/6J mice deficient in stimulator of interferon genes (STING), a downstream adaptor molecule of cytoplasmic DNA sensor cyclic GMP-AMP synthase (cGAS). STING^−/−^ mice showed an elevated response at a low dose (2 × 10^10^ vg) and a somewhat reduced response at a high dose (2 × 10^11^ vg) when compared to WT C57BL/6J mice. These differences were found to be statistically non-significant (Supplementary Figure S3). Therefore, signaling through STING in response to cytoplasmic DNA sensing may not be required for CD8^+^ T-cell responses against the transgene product. Interestingly, C57BL/6J mice deficient in TBK1 (TANK binding kinase 1), which integrates TLR3-TRIF, MDA5/RIG-I-MAVS, and cGAS-STING signaling pathways lacked responses at both vector doses (Figure 3B,C).

### 3.4. Simultaneous Block of Two Innate Pathways Indicates Redundancy at High-Vector Doses and Provides Path for Targeted Therapeutic Intervention

**Figure 4:**
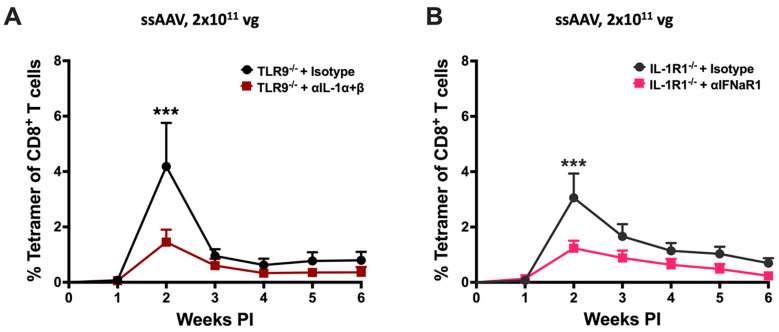
Combined inhibition of TLR9 and IL-1 signaling leads to an abrogated cellular response to AAV-encoded transgene product in muscle. (**A**) Kinetics of OVA-specific CD8^+^ T-cell response in TLR9^−/−^ mice following intramuscular gene transfer with a high dose (2 × 10^11^ vg) of the ssAAV1 vector. TLR9^−/−^ mice were treated with an isotype control antibody (black line) or a combination of anti-IL-1α and anti-IL-1β antibodies (brown line). (**B**) Kinetics of OVA-specific CD8^+^ T-cell response in IL-1R1^−/−^ mice following intramuscular gene transfer with a high dose (2 × 10^11^ vg) of the ssAAV1 vector. IL-1R1^−/−^ mice were either treated with an isotype control antibody (charcoal black line) or with anti-IFNaR1 antibody (pink line). Each experimental group consisted of five mice. Data are presented as mean ± SEM. Significant differences between WT and knockout mice are denoted as follows: the level of significance is indicated as *** *p* < 0.001. Non-significant differences are not indicated.

A logical next step based on our findings was to target two pathways when performing high-dose gene transfer. Given that vector engineering strategies are available to reduce TLR9 signaling and a drug to block the IL-1R1 is already approved and in clinical use in the form of a recombinant human IL-1R antagonist, we focused on these two pathways. For initial proof-of-principle, we used a combination of knockout mice and antibody treatment (Figure 4). TLR9^−/−^ mice were treated with a combination of α-IL-1α and α-IL-1β antibodies, starting 1 day before IM administration of 2 × 10^11^ vg of the ssAAV1-CMV-OVA. Alternatively, IL-1R1^−/−^ mice were treated similarly with α-IFNaR (to block type I IFN signaling downstream of TLR9 and other pathways). In both experiments, the frequency of induced OVA-specific CD8^+^ T cells was significantly reduced (~3-fold at the 2-week peak response) compared to mice that received isotype control antibody (Figure 4A,B). In conclusion, disruption of two signaling pathways reduces CD8^+^ T-cell responses against the transgene product at high-vector doses.

**Figure 5:**
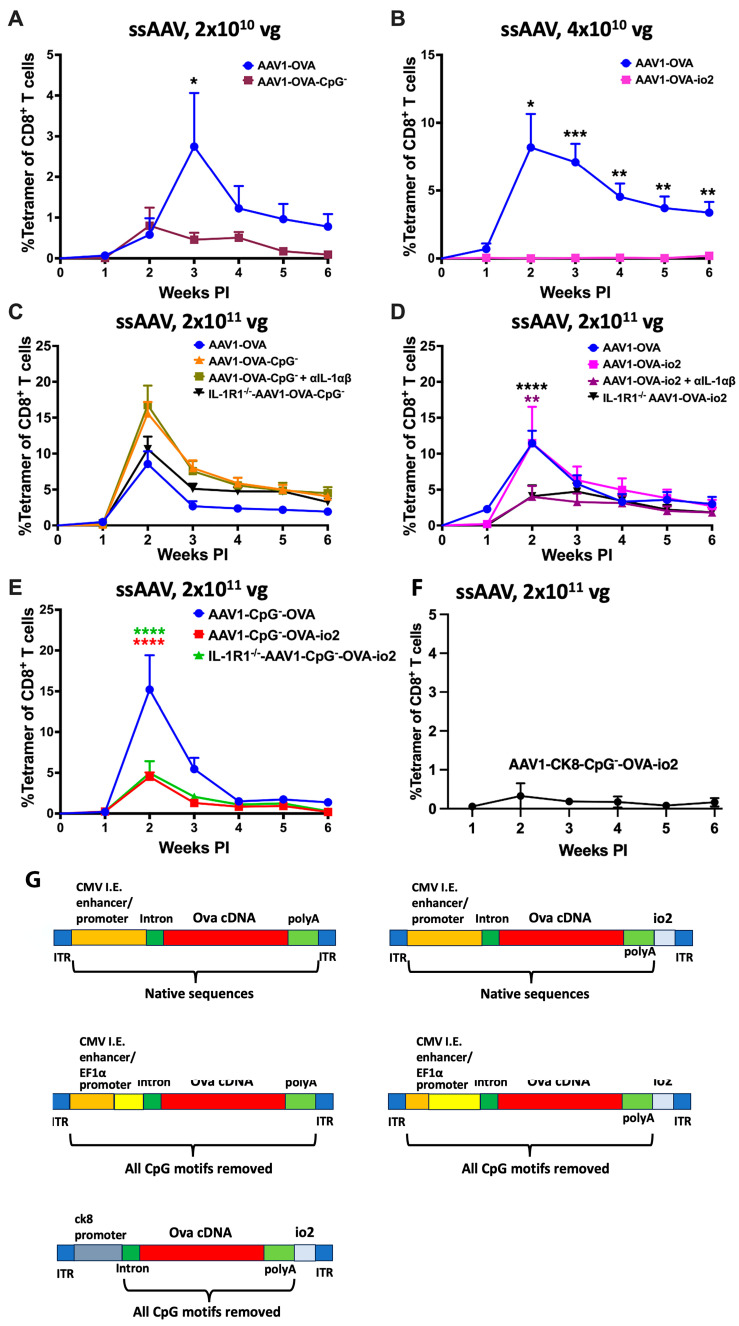
The TLR9 inhibitory sequence (io2), not CpG depletion, along with the IL-1 blockade, abrogates the cellular response to the AAV-encoded transgene product in muscle. (**A**) Kinetics of OVA-specific CD8^+^ T-cell response in C57BL/6J − WT mice following intramuscular gene transfer with low dose (2 × 10^10^ vg) of CpG^+^ (blue line) or CpG^−^ (plum line) ssAAV1 vector. (**B**) Kinetics of OVA-specific CD8^+^ T-cell response in C57BL/6J − WT mice following intramuscular gene transfer with low dose (4 × 10^10^ vg) of the ssAAV1 vector with (magenta line) or without (blue line) TLR9 inhibitory sequence (io2). (**C**) Kinetics of OVA-specific CD8^+^ T-cell response in C57BL/6J − WT (blue, orange and light green lines) and IL-1R1^−/−^ (black line) mice following intramuscular gene transfer with a high dose (2 × 10^11^ vg) of CpG^+^ (blue line), CpG^−^ (orange, light green, and black line). In two of the three groups (black, orange, and light green lines) of mice that received the CpG^−^ AAV1 vector, IL-1 signaling was inhibited either by using IL-1R1^−/−^ mice (black line) or with anti-IL-1α and anti-IL-1β antibodies (light green line). (**D**) Kinetics of OVA-specific CD8^+^ T-cell response in C57BL/6J − WT (blue, magenta, and purple lines) and IL-1R1^−/−^ (black line) mice following intramuscular gene transfer with a high dose (2 × 10^11^ vg) of the ssAAV1 vector with (black, magenta, and purple line) or without (blue line) TLR9 inhibitory (io2) sequence. In two of the three groups (black, magenta, and purple line) of mice that received an AAV1 vector containing TLR9 inhibitory (io2) sequence, IL-1 signaling was inhibited either by using IL-1R1^−/−^ mice (black line) or with anti-IL-1α and anti-IL-1β antibodies (purple line). (**E**) Kinetics of OVA-specific CD8^+^ T-cell response in C57BL/6J − WT (blue and red line) and IL-1R1^−/−^ (green line) mice following intramuscular gene transfer with a high dose (2 × 10^11^ vg) of the CpG^−^ ssAAV1 vector with (red and green line) or without (blue line) TLR9 inhibitory (IO2) sequence. IL-1R1^−/−^ mice (green line) were used to test the combined effect of TLR9 (CpG depletion and TLR9 inhibitory sequence) and IL-1 inhibition on transgene product-specific CD8^+^ T-cell response following AAV muscle gene transfer. (**F**) OVA-specific CD8^+^ T-cell response in C57BL/6J − WT mice following intramuscular gene transfer with a high dose (2 × 10^11^ vg) of the ssAAV1-ck8-CpG^−^-OVA-io2 vector. (**G**) Cartoon depicting the arrangement of AAV expression cassettes with native, CpG^−^ and/or TLR9 inhibitory (io2) sequence of AAV vectors used in the present study. Each experimental group consisted of five mice. Data are presented as mean ± SEM. Significant differences between control and experimental mice are denoted with *. In Figure D, significance is shown between WT mice-AAV1-OVA (blue line) and WT mice–AAV1-OVA-io2 + anti-IL-1α and anti-IL-1β (purple line) and between WT mice-AAV1-OVA (blue line) and IL-1R1^−/−^ mice–AAV1-OVA-io2 (black line). The level of significance is indicated as * *p* ≤ 0.05, ** *p* < 0.01, *** *p* < 0.001, and **** *p* < 0.0001. Non-significant differences are not indicated.

To further explore this approach, we constructed vector genomes with reduced TLR9 activation. The ssAAV1-OVA-CpG^−^ vector contains an OVA expression cassette that entirely lacks CpG motifs (containing CMV enhancer, EF1α promoter, and CpG-depleted OVA cDNA; analogous to published constructs expressing LacZ or human factor IX; Figure 5G and Table 1) [22,29]. At the low dose (2 × 10^10^ vg), the response in WT mice was significantly lower compared to the CpG^+^ ssAAV1-CMV-OVA vector (80 CpG motifs) that we had used in all prior experiments (Figure 5A). However, at the high dose (2 × 10^11^ vg), the ssAAV1-OVA-CpG^−^ vector elicited slightly higher CD8^+^ T-cell responses than CpG^+^ ssAAV1-CMV-OVA, albeit this did not reach statistical significance (Figure 5C). While treatment with α-IL-1α and α-IL-1β failed to reduce the response to high-dose ssAAV1-OVA-CpG^−^, IL-1R1^−/−^ mice showed a somewhat reduced response, which was, however, not lower than the response to conventional (i.e., CpG^+^) ssAAV1-CMV-OVA vector in WT mice (Figure 5C).

In an alternate approach to block TLR9 activation, we constructed a vector (ssAAV1-OVA-io2), in which the TLR9 inhibitory sequence io2 was added downstream of the OVA cDNA in the original CpG^+^ ssAAV1-CMV-OVA vector (Figure 5F) [33]. Impressively, no response was observed at a dose of 4 × 10^10^ vg (Figure 5B). However, this effect was again lost at the high (2 × 10^11^ vg) vector dose (Figure 5D). In contrast to our experience with the CpG^−^ vector, IL-1 signaling blockade using either α-IL-α and α-IL-1β treatment in WT or performing gene transfer in IL-1R1^−/−^ mice significantly reduced the CD8^+^ T-cell response against OVA after high-dose ssAAV1-OVA-io2 administration (Figure 5D). Subsequently, we concorporated the io2 sequence into the CpG^−^ vector to create ssAAV1-CpG^−^-OVA-io2 (Figure 5G). The addition of io2 resulted in a highly significant, 3-fold reduction in the CD8^+^ T-cell response against OVA in WT mice and also reduced, albeit to a lesser extent, the response in TLR9^−/−^ mice (Figure 5E and Supplementary Figure S4). However, as with ssAAV1-OVA-CpG^−^ parent vector, IL-1 blockade failed to further reduce the response (Figure 5E). In contrast, replacement of the ubiquitous enhancer/promoter sequence in this construct with the muscle-specific ck8 promoter, as utilized in our recent canine study, reduced the CD8^+^ T-cell response to nearly undetectable at the high (2 × 10^11^ vg) vector dose (Figure 5F) [44]. Moreover, robust transduction of muscle in WT mice was sustained for at least 8 weeks with this vector (which combines CpG depletion, io2, and tissue-specific promoter). However, modest CD8^+^ T-cell infiltration was still observed (Figure 2C). This is in sharp contrast to muscles from mice that had received the original CpG^+^ ssAAV1-CMV-OVA vector and mostly lacked OVA expression and CD8^+^ T-cell infiltration at the 8-week time point (Figure 2C), indicating that transgene expression was lost, and the local response had mostly resolved. The few areas with scarce residual OVA-expressing fibers, which were found in 3/3 WT mice, were targeted by CD8^+^ T cells (Figure 2C).

### 3.5. Effect of Innate Immune Pathways on Antibody Formation against Transgene Product

**Figure 6:**
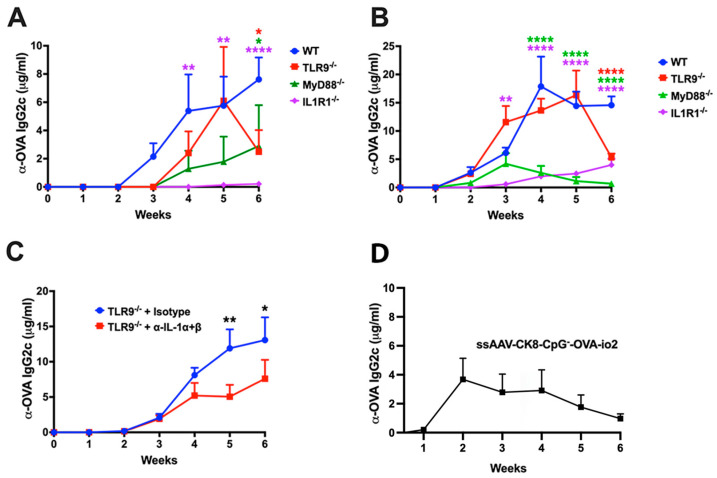
Minimal effect of innate immune blockade on transgene product-specific antibody formation following AAV1 muscle gene transfer. (**A**,**B**) Time course of OVA-specific IgG2c antibodies following low (2 × 10^10^ vg; (**A**)) and high (2 × 10^11^ vg; (**B**)) dose administration of the ssAAV1 vector in C57BL/6J − WT and knockout mice lacking a specific innate sensor. (**C**) OVA-specific IgG2c antibodies following high (2 × 10^11^ vg) dose of the ssAAV1 vector in TLR9^−/−^ mice, which were either treated with isotype control antibody (blue line) or a combination of anti-IL-1α and anti-IL-1β antibodies (red line). (**D**) Antibody formation against OVA in C57BL/6J − WT injected with ssAAV1-ck8-CpG^−^-OVA-io2 vector. Each experimental group consisted of five mice. Data are presented as mean ± SEM. The level of significance between WT and knockout mice is indicated as * *p* ≤ 0.05, ** *p* < 0.01, and **** *p* < 0.0001. Non-significant differences are not indicated.

In some of the experiments that explored the roles of TLR9 and IL-1R1 (and their common cytoplasmic adaptor MyD88), we also assessed antibody formation against OVA in select experimental groups injected with ssAAV1-CMV-OVA. IgG2c typically constitutes the dominant subclass in C57BL/6J mice in response to OVA. C57BL/6J mice produced IgG2c against OVA (starting at week 3) at a vector dose of 2 × 10^10^ vg (Figure 6A). This response was delayed by 1 week in mice deficient in MyD88 and TLR9 (Figure 6A). Responses in the knockout mice were generally weaker than in WT mice, except for TLR9^−/−^, which reached a maximum titer similar to that of WT mice. IgG2c formation in IL-1R1^−/−^ mice was only detectable at low titer by weeks 5–6 (Figure 6A). At the high dose (2 × 10^11^ vg), WT mice showed IgG2c formation against OVA within 2 weeks, and titers increased by week 4 to levels 2–4-fold higher than for the low dose (Figure 6B). Mice deficient in MyD88, or IL-1R1, produced substantially lower titers, while TLR9^−/−^ mice, except for the week 6 time point, had titers comparable to WT mice (Figure 6B). The IL-1 blockade in TLR9^−/−^ mice that received 2 × 10^11^ vg lowered IgG2c formation against OVA by ~2-fold (Figure 6C). In all high-dose cohorts, every experimental group developed peak titers of at least 5 μg/mL. Overall, the effect of innate immune blockade on antibody formation against the transgene product was modest. Similarly, the replacement of the ubiquitous enhancer/promoter with the muscle-specific ck8 promoter in the ssAAV1-CpG^−^-OVA-io2 construct (albeit very effective in limiting CD8^+^ T-cell responses) still resulted in substantial antibody formation against OVA (Figure 6D).

## 4. Discussion

Similar to responses against viral pathogens, anti-viral vector CD8^+^ T-cell responses are triggered by innate immune signals, which may be derived from sensing of PAMPs featured by the viral vector particles (e.g., capsid or genome) or the infected tissue (damage-associated molecular patterns, which may derive from the viral infection of the vector administration method). Key cellular components in response to AAV vectors include pDCs (which sense the viral DNA genome via the endosomal receptor TLR9 and are able to infiltrate the infected tissue), cDCs (which respond to cytokine conditioning by pDCs and present antigen via MHC-I to CD8^+^ T cell and via MHC-II to CD4^+^ T cells), and CD4^+^ T helper cells (which provide co-stimulation) [19,20,21]. IFNα/β is produced by pDCs upon TLR9 stimulation, while pDCs may also produce IL-1α/β during TLR9-independent responses [20,21,25]. A complex picture emerges while attempting to identify innate response pathways in skeletal muscle that are critical for the induction of CD8^+^ T-cell responses to AAV-encoded transgene products. At lower vector doses, disruption of a single innate signaling pathway substantially reduces the response, while two or more pathways must be blocked at higher doses of the ssAAV, indicating redundancy in stimulating pathways (including sensing of IL-1, DNA, and dsRNA).

Further, we show that alternative methods to TLR9 blockade (depletion of CpG motifs and inclusion of TLR9i sequences) can be combined. However, surprisingly, not every vector construct responded to the IL-1 blockade. We also find that responses to scAAV, which we previously reported to more strongly direct TLR9 signaling and drive more destructive CD8^+^ T-cell responses, are more TLR9 dependent even at higher vector doses, suggesting a skewing of innate signaling toward TLR9 and downstream pathways for scAAV vectors [34,58].

While optimal, CD8^+^ T-cell activation appears to depend on multiple innate pathways to be intact at low-vector doses, likely acting in concert; the ability to dampen CD8^+^ T-cell responses against the transgene product by avoiding or blocking innate signaling pathways is limited at high doses. While we experienced variability in the OVA-specific CD8^+^ T cell frequencies in WT mice between experiments, a vector dose of 2 × 10^11^ vg consistently resulted in a more rapid response that peaked at 2 weeks, while a ten-fold lower vector dose induced a response that peaked at 3 weeks. At the high dose, a blockade of a single pathway had little effect, while a blockade of two pathways typically reduced but did not entirely eliminate the response. Mice deficient in MyD88 or TBK1, which are critical components of multiple signaling pathways, had low or no responses upon high-dose gene transfer, consistent with the notion that multiple innate signaling pathways contribute redundant activation signals. However, the scAAV vector, in contrast to ssAAV, showed a substantially weaker response in TLR9^−/−^ mice even at a high-vector dose; a residual CD8^+^ T-cell response indicates that additional activation signals exist regardless of the vector genome configuration.

Others have shown that AAV vectors may generate double-stranded RNA (dsRNA) upon transduction through inherent promoter activity in the inverted terminal repeats (ITRs) [59]. If exported into the cytoplasm, this dsRNA could be sensed by MDA5 and RIG-I, thereby inducing type I IFN expression as was shown in hepatocytes. Whether these events link to adaptive immune responses is unknown. We find some evidence for the involvement of dsRNA by the TLR3-TRIF pathway (but not MDA5 or RIG-I) in CD8^+^ T-cell activation upon muscle gene transfer. While experimental results in both TLR3 and TRIF-deficient mice support a contributing role for this pathway, the differences in the timeline and degree of reduction of the responses (likely at least in part caused by the differences in background strains) prevent us from making solid conclusions about the robustness of the contribution relative to other pathways. Contribution of the endosomal receptor TLR3, which is expressed by cDCs (but not pDCs), may not necessarily reflect expression from the AAV vector but uptake of dsRNA released by damaged cells, a known potent DAMP [60]. Other DAMPs not investigated here, such as heat shock proteins, may also play a role. It is possible that the IM route, which causes some level of tissue damage at the injection site, increases the likelihood of generating DAMPs. Local antigen presentation plays an important role in B- and T-cell activation in skeletal muscle, as we have previously shown, which prompted a limit on the dose per injection site in a muscle-directed AAV gene therapy trial for hemophilia B [61,62,63].

Two innate/cytokine signaling pathways have previously been identified to link to CD8^+^ T-cell activation, namely TLR9 > MyD88 > type I IFN > IFNaR [20,21] and IL-1α,β > IL-1R1 > MyD88 [25]. In both cases, the critical cytokine is expressed by pDCs. The IL-1R1 pathway was specifically identified as a requisite for TLR9-independent CD8^+^ T-cell responses against the transgene product at low dose gene transfer to the liver, resulting in CD8^+^ T cell in the hepatic microenvironment, while higher vector doses resulted in immune tolerance induction [46]. This contrasts with the more rapid and potent responses seen in muscle-directed gene transfer. Here, we provide multiple pieces of evidence for diminished CD8^+^ T-cell responses to high-dose muscle gene transfer if both TLR9 and IL-1 signaling are targeted. For example, the addition of the TLR9i sequence io2 combined with IL-1 blockade (genetic or antibody-mediated) was effective, albeit it could not entirely prevent the response. Curiously, the CpG-depleted vector failed to be influenced by IL-1 blockade, at least at high doses. The addition of io2 reduced the response to the high-dose CpG^−^ vector in WT mice, which could not be further reduced again by IL-1 blockade. There is no obvious reason why this construct induces CD8^+^ T-cell responses in an IL-1-independent fashion—a phenomenon that requires further study. It is possible that the combination of CMV enhance/EF1α promoter in the CpG-depleted construct is more active in antigen-presenting cells. However, this would not alleviate the need for activation signals or cytokine stimulation of DCs for T-cell activation.

Importantly, our results demonstrate that CpG depletion and TLR9i approaches can be combined to reduce T-cell responses. At a high dose, the ssAAV1-OVA-CpG^+^, ssAAV1-OVA-CpG^−^, and ssAAV1-OVA-io2 vectors produced similarly potent CD8^+^ T-cell responses against OVA in WT mice. Success in reducing the response in WT mice injected with the high dose of the combination vector ssAAV1-CpG^−^-OVA-io2 would not have been predicted based on the results with ssAAV1-OVA-CpG^+^ in TLR9-deficient mice. It is possible that io2 further reduced TLR9 signaling that may occur in response to CpG motifs in the ITRs or to accidentally packaged plasmid backbone sequences. However, this outcome also raises the possibility that io2 has additional effects besides TLR9 inhibition, as has been speculated by others [33,64]. The modest effect in TLR9-deficient mice in reducing the response to ssAAV1-OVA-CpG^−^ is consistent with but does not prove this conclusion. Although experiments in STING-deficient mice did not reveal an effect of cytoplasmic DNA sensing, we cannot rule out a role for io2 in blocking a DNA or other innate sensor yet to be identified. It is possible that these knockout mice have skewed responses due to the upregulation of other/compensatory pathways, as perhaps suggested by the somewhat increased or decreased responses at different vector doses relative to those in WT mice.

In previous studies, we found no evidence for TLR2 involvement in CD8^+^ T-cell responses to AAV capsid or transgene product in muscle or liver-directed gene transfer and, therefore, did not conduct further studies of this receptor here [20,25,35]. However, others found evidence for sensing of AAV capsid by TLR2 of human liver macrophages and developed a strategy to incorporate a TLR2 inhibitory peptide into the capsid [65]. As we cannot entirely rule out a role for the innate sensing of a capsid as another activation signal, this strategy might be tested in muscle gene transfer.

## 5. Conclusions

**Figure 7:**
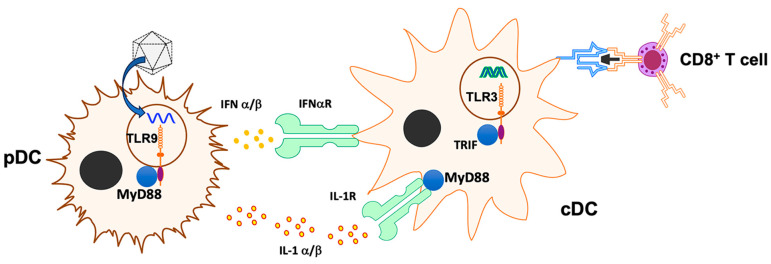
Model depicting innate sensors, cytokines, and cell types involved in activation of CD8^+^ T-cell response following muscle gene transfer. The illustration depicts previously identified cells and molecules, and putative molecules identified in the present study.

Our findings have multiple mechanistic and therapeutic implications. Intramuscular AAV vector administration triggers multiple innate immune pathways that promote CD8^+^ T-cell activation against the transgene product (Figure 7). A blockade of any of these weakens the T-cell response at lower vector doses, while redundancy of activation signals requires targeting at least two pathways at higher vector doses, supporting clinical observations that high-vector doses pose a greater risk for immunotoxicities. The IL-1R1 pathway recently identified to promote CD8^+^ T-cell responses in hepatic AAV gene transfer is also active in skeletal muscle, where it is similarly crucial as TLR9. This scenario is distinct from low-dose liver gene transfer, where IL-1R1 deficiency but not TLR9 deficiency abrogates CD8^+^ T-cell activation against the transgene product [25]. In the context of muscle gene transfer, IL-1R1 appears to also have a role in the transition of transgene product-specific CD8^+^ T cells into a memory phenotype. The IL-1R1 receptor has multiple ligands, and its regulation is complex. Hence, these roles may not only reflect the sensing of IL-1 cytokines but also, for example, regulation by the IL-1R antagonist.

High-vector doses, efficient gene transfer, and strong promoters, among other factors, increase levels of the expressed antigen, which is expected to increase antigen presentation to T cells. In this study, we used an antigen that contains a strong CD8^+^ T cell epitope. Which vector dose constitutes a high or low dose for immune responses varies for different transgene products and hosts, depending on the number and strength of T-cell epitopes. For instance, while the delivery of full-length dystrophin may provide superior muscle strength compared to micro-dystrophins, they also contain a larger set of potential T cell epitopes [66,67]. In a prior study on IM F9 gene transfer in hemophilia B mice, we found that the CpG-depleted vector (using the same enhancer/promoter elements as here) substantially reduced but again did not eliminate CD8^+^ T-cell responses (using a similar AAV1 vector dose as the high dose in this study with the OVA transgene) [22]. Innate immunity triggered by AAV genomes with high CpG contents may cause diverse treatment complications. Examples include enhanced CD8^+^ T-cell responses to capsids, loss of F9 gene expression in AAV-transduced livers of hemophilia B patients, or inference with neuronal structure and function upon CNS gene transfer as recently shown in a mouse model [20,22,29,30,31,32,38,68]. Our study supports that the combination of CpG depletion and the inclusion of TLR9i sequences could be further explored to minimize these effects.

The sum of our findings shows that, albeit helpful, it is difficult to entirely prevent destructive T-cell responses in muscle gene transfer by targeting innate immune pathways. Additional vector engineering and immune modulation strategies should be employed. While the use of muscle-specific promoters has not always yielded the desired effect, the inclusion of micro-RNA targets for elimination of the transgene’s transcript in DCs may be helpful, for example [69,70,71]. Nonetheless, our study shows encouraging results when including a muscle-specific promoter in an expression cassette that is not only CpG-depleted but also contains a sequence that inhibits TLR9 (and possibly additional pathways). The ck8 promoter introduces nine CpG motifs, which may, however, be counteracted by the inclusion of io2 (and still represents an ~1-log reduction in CpG contents compared to the original vector). Diseases with inflamed muscle, such as muscular dystrophies, are likely to supply additional tissue-derived DMAPs, which may necessitate additional immune suppression [13,72].

## Figures and Tables

**Table 1:** Effect of vector genome engineering and IL-1 blockade on CD8^+^ T response against OVA upon ssAAV1-OVA vector IM administration.

Vector Dose	Low	High
IL-1 blockade	↓	-
TLR9i (io2)	↓	-
TLR9i (io2) + IL-1 blockade	nd	↓
CpG depletion	↓	-
CpG depletion + IL-1 blockade	nd	-
CpG depletion + TLR9i (io2)	nd	↓
CpG depletion + TLR9i (io2) + IL-1 blockade	nd	↓ (but not lower than CpG depletion + TLR9i without IL- blockade)
CpG depletion + TLR9i (io2) + muscle-specific promoter	nd	↓↓

↓ indicates reduced response compared to standard ssAAV-CMV-OVA vector given without immune modulation (↓↓ indicates most robustly reduced response); - indicates no effect on the magnitude of response; nd—experiment not done.

## Data Availability

The raw data supporting the conclusions of this article will be made available by the authors on reasonable request.

## Supplementary Materials

The following supporting information can be downloaded at: https://www.mdpi.com/article/10.3390/v16101507/s1, Figure S1: Analysis of transgene product-specific CD8+ T cell response in peripheral blood of mice transduced with AAV1-OVA vectors; Figure S2: OVA specific CD8+ T cell response in C57BL/6NJ mice deficient in RIG-I as a function of vector dose; Figure S3: OVA specific CD8+ T cell response in mice deficient in cytoplasmic DNA sensing; Figure S4: OVA specific CD8+ T cell response in mice deficient in TLR9 and transduced with CpG depleted vector.
