# Supplementary material for: Redundancy in Innate Immune Pathways That Promote CD8+ T-Cell Responses in AAV1 Muscle Gene Transfer
Source: Viruses. 2024 Sep 24;16(10):1507. doi: 10.3390/v16101507 (PMC11512359; doi:10.3390/v16101507)
Supplement: Supplementary file 1 [file viruses-16-01507-s001.zip › viruses-3161458-supplementary.pdf]

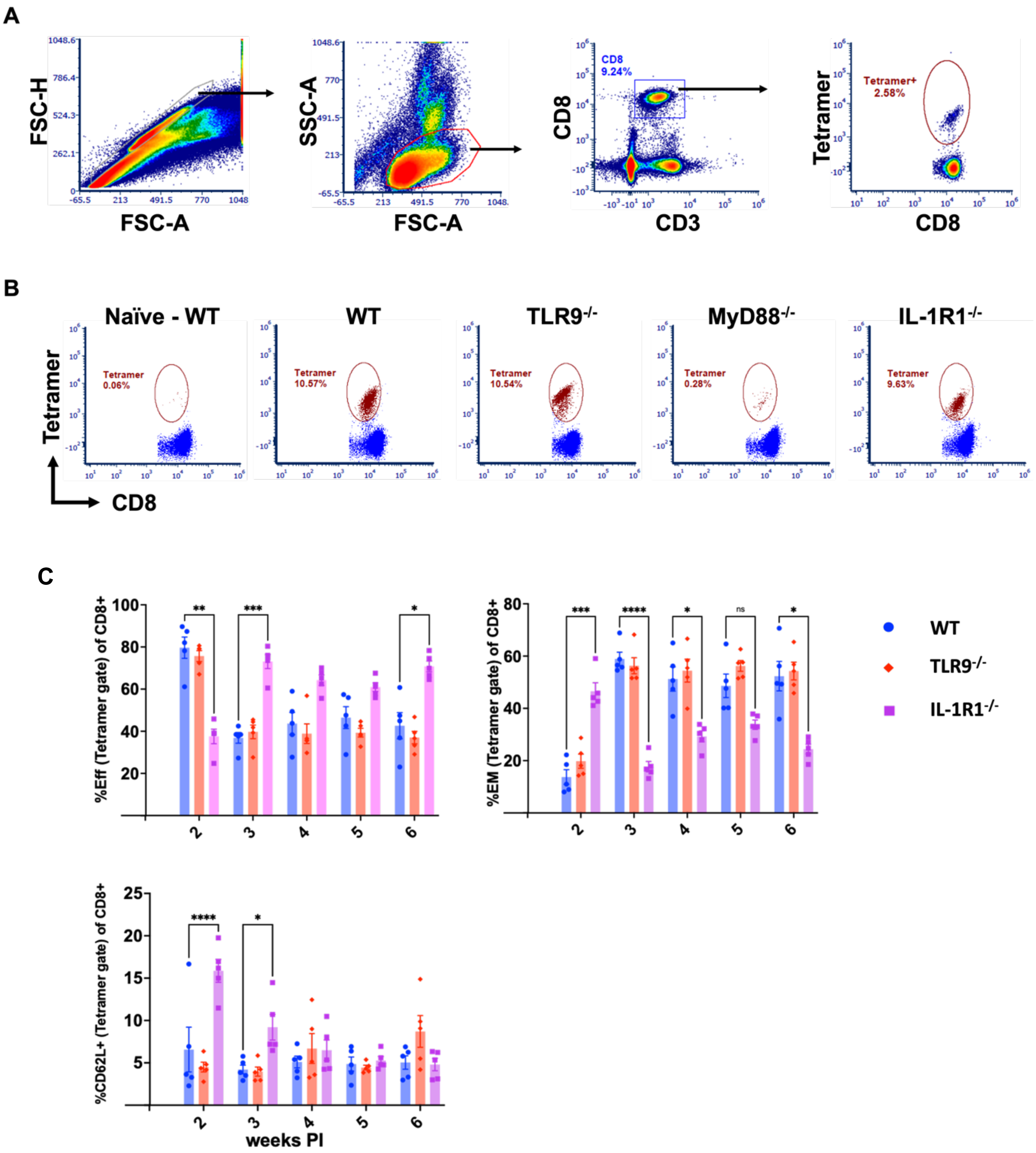

Figure S1: Analysis of transgene product-specific $CD8^+$ T cell response in peripheral blood of mice transduced with AAV1-OVA vectors. A) Gating scheme for OVA-specific $CD8^+Tetramer^+$ T cells. **B)** Representative dot plots showing OVA specific $CD8^+$ T cells in WT, $TLR9^{-/-}$, $MyD88^{-/-}$ and $IL\text{-}1R1^{-/-}$ mice 2 weeks after IM administration of $2x10^{11}$ vg ssAAV1-CMV-OVA. **C)** Phenotyping of OVA-specific $CD8^+Tetramer^+$ T cells in C57BL6/J – WT, $TLR9^{-/-}$ and $IL\text{-}1R1^{-/-}$ mice at different time points following AAV administration (n=5/experimental group). Phenotyping was based on the expression of cell surface markers CD44, CD62L, and CCR7. Gating scheme used for the phenotyping was as previously described (Kumar *et al. Mol Ther* **25:** 880-891, 2017).

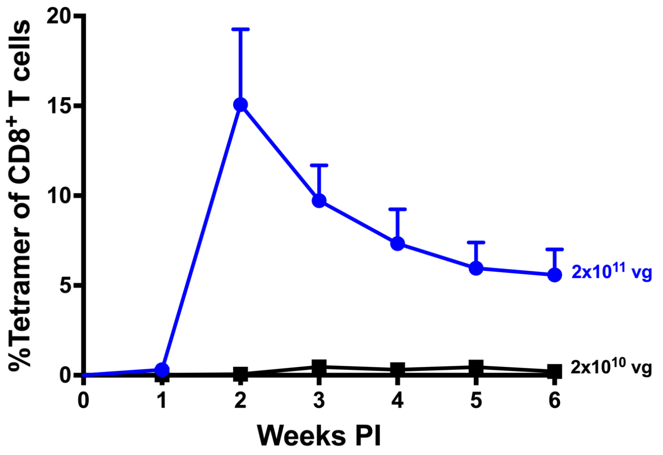

Figure S2: OVA specific CD8$^+$ T cell response in C57BL/6NJ mice deficient in RIG-I as a function of vector dose.

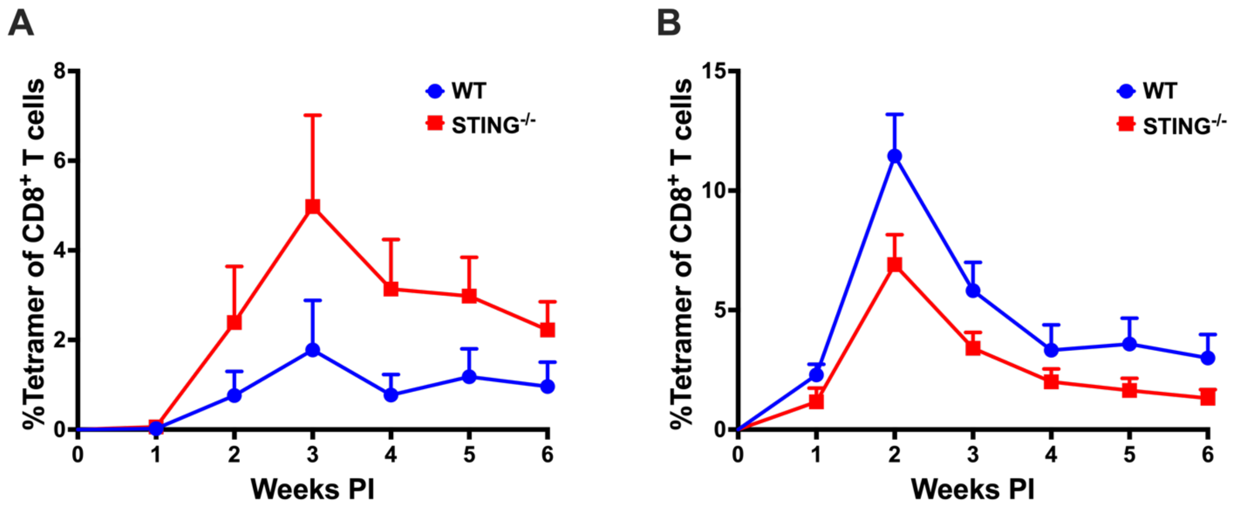

Figure S3: OVA specific CD8$^+$ T cell response in mice deficient in cytoplasmic DNA sensing. A & B) Kinetics of OVA specific CD8$^+$ T cell response in C57BL6/J - WT, and STING$^{-/-}$ mice following intramuscular gene transfer with 2x10^{10} vg (**A**) or 2x10^{11} vg (**B**) of ssAAV1-CMV-OVA vector (n=5/experimental group).

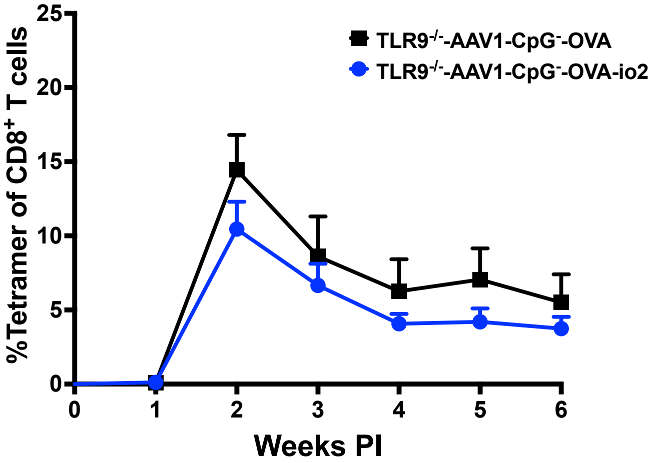

Figure S4: OVA specific CD8$^+$ T cell response in mice deficient in TLR9 and transduced with CpG depleted vector. Kinetics of OVA specific CD8$^+$ T cell response following intramuscular gene transfer with 2x10^{11} vg of ssAAV1-CpG$^-$-OVA or ssAAV1-CpG$^-$-OVA-io2 vector in TLR9$^{-/-}$ mice (n=5/experimental group).
